# Editorial: Digital tools for relaxation and stress management: use, effectiveness and implementation

**DOI:** 10.3389/fdgth.2024.1366065

**Published:** 2024-01-31

**Authors:** Sylvie Bernaerts, Philip Lindner

**Affiliations:** ^1^ Psychology and Technology, Centre of Expertise Care and Well-Being, Thomas More University of Applied Sciences, Antwerp, Belgium; ^2^ Department of Clinical Neuroscience, Centre for Psychiatry Research, Karolinska Institutet & Stockholm Health Care Services, Stockholm, Sweden

**Keywords:** mental health, digital mental health, digital tools, stress management, relaxation

**Editorial on the Research Topic** Digital tools for relaxation and stress management: use, effectiveness and implementation

Stress, depression, and anxiety remain top contributors to the global burden of disease (1), with signs of increasing prevalence after societal challenges such as the COVID-19 pandemic (2), political polarization (3), a struggling economy (4), climate change (5), and more. With the increasingly ubiquitous integration of technology into our everyday lives, digital tools have been an attractive vector to disseminate evidence-based interventions to affected individuals. Digital mental health, or the use of technology in mental health care, includes technology varying from online platforms and websites, over chatbots, mobile health applications and wearables, to extended reality, with artificial intelligence on the way. These tools can be used as self-help, in blended care or fully guided (depending on the respective tool). These tools have great potential to reach hard-to-reach individuals, either due to remoteness or stigma, to help individuals on waiting lists, and to make providing care more efficient for clinicians. For example, in low- and middle-income countries digital mental health interventions for depression and anxiety in adults have shown moderate to high effectiveness in reducing symptoms (6). As another example, virtual reality (VR) is an effective tool for exposure in the treatment of anxiety disorders (7).

Although hardly a novel area of research (8), constantly evolving technology—and usage thereof—requires new research on how to best make use of the inherent capabilities of technology for mental health purposes, including potent relaxation and stress management tools.

The aim of the Research Topic *Digital tools for relaxation and stress management: use, effectiveness and implementation* was to attract and collect recent research on this area of great public health potential. The final Research Topic includes original research, a clinical trial, an opinion piece and a case report.

In a secondary analysis of an RCT, Küchler et al. examined mediators and moderators of the antidepressant effects of a digital, web-based, seven-module mindfulness intervention for college students. Of the examined potential mediators, only mindfulness was found to mediate depression scores, while number of semesters was a moderator of depression scores.

In another study targeting university students, Kaligis et al. report the findings of a pragmatic RCT of a four-module digital, web-based intervention designed to increase psychological resilience among medical students. Over the twelve-week follow-up period, a significant time × group interaction effect revealed an increase in resilience compared to the control.

In their opinion piece, Kaleva and Riches discuss the potential of combining VR technology—which in recent years have developed into an off-the-shelf consumer technology (9)—with content designed to trigger an Autonomous Sensory Meridian Response (ASMR) (10) evoking a pleasant bodily sensations and thereby increasing wellness. Given the popularity of ASMR content on social media (suggesting real-world effectiveness), and the capacity of VR to deliver immersive experiences, the authors call for more research on this promising topic.

In a case report, Woo and Lee describe the use of a novel VR relaxation protocol applied to palliative cancer care, that included a personally selected 360° nature environment and manualized verbal ques and questions anchored in e.g., self-determination theory.

Finally, Kim et al. report the outcomes of an experiment designed to uncover the impact of cybersickness on *in virtuo* anxiety. Using a randomized cross-over design, the authors showed that anxiety and skin conductivity saw a greater increase when watching a less dizzy video after having first watched a dizzier video, than vice versa. These findings have obvious design implications for VR relaxation interventions, that are popular on application stores (11) and even implemented in routine psychiatric care (12).

The five above-mentioned articles cover a diverse set of novel digital initiatives to combat stress and other mental health problems, applied to different setting and with different target groups. Together, they showcase the ever-expanding topic of digital tools for relaxation and stress management, not only adding important insights to the extant literature, both also suggesting research questions that should be examined in future studies.
